# Analysis of cyt0kine gene expression in stimulated T cells of small children by semi-quantitative PCR

**DOI:** 10.1155/S0962935195000329

**Published:** 1995-05

**Authors:** H. Koning, M. R. M. Baert, R. de Groot, H. J. Neijens, H. F. J. Savelkoul

**Affiliations:** 1 Department of Immunology Erasmus University Rotterdam The Netherlands; 2 Department of Pediatrics Sophia Children's Hospital Rotterdam The Netherlands

## Abstract

Only limited amounts of peripheral blood samples can be obtained from small children. Therefore, a polymerase chain reaction (PCR) aided analysis of cytokine gene expression by PBMC or T cells is a valuable tool. We present a combination of procedures to obtain an accurate estimation of the expression of the cytokines IL-4 and IFN-γ. This can be performed on T cells purified from blood samples of up to 5 ml in volume from children aged 0–4 years with allergic asthma and atopic dermatitis. This procedure includes multiple sampling of PCR products to determine the linear phase of the PCR; inter-experiment correction using a helper T-cell clone, expressing both IL-4 and IFN-γ; interpatient correction by comparing the expression of a housekeeping gene (HPRT); and finally the development of specific software to analyse densitometric data obtained by scanning photographs of agarose gels, separating PCR products. In this way it is possible to study cytokine gene expression from a very small amount of material.


Mediators of Inflammation 4, 196–204 (1995)

### Analysis of cytokine gene expression in stimulated T cells of small children by semi-quantitative PCR

H. Koning,[1,2,CA] M. R. M. Baert,[1,2] R. de Groot,[2] H. J. Neijens[2] and H. F. J. Savelkoul[1]

[1]Department of Immunology, Erasmus University, Rotterdam;
[2]Department of Pediatrics, Sophia Children's Hospital, Rotterdam, the Netherlands

[CA]Corresponding Author

**ONLY limited amounts of peripheral blood samples can be obtained from small children. Therefore, a polymerase chain reaction (PCR) aided analysis of cytokine gene expression by PBMC or T cells is a valuable tool. We present a combination of procedures to obtain an accurate estimation of the expression of the cytokines IL-4 and IFN-γ. This can be performed on T cells purified from blood samples of up to 5 ml in volume from children aged 0–4 years with allergic asthma and atopic dermatitis. This procedure includes multiple sampling of PCR products to determine the linear phase of the PCR; inter-experiment correction using a helper T-cell clone, expressing both IL-4 and IFN-γ; inter-patient correction by comparing the expression of a housekeeping gene (*HPRT*); and finally the development of specific software to analyse densitometric data obtained by scanning photographs of agarose gels, separating PCR products. In this way it is possible to study cytokine gene expression from a very small amount of material.**

**Key words:** Children, Cytokine, PCR, Small volumes, T cells

### Introduction

Allergic diseases such as allergic asthma and atopic dermatitis are generally characterized by increased IgE levels in the serum.[1–3] Cytokines play an important role in the regulation of IgE synthesis.[4] IL-4 is an inducer of IgE synthesis, while IFN-γ suppresses IgE synthesis.[5,6] Other cytokines play an additional role in the regulation of IgE. IL-5 has an enhancing effect on the stimulatory activity of IL-4[7] and IL-10 has a down-modulating effect on the production of IFN-γ.[8] IL-13 is a recently discovered cytokine, produced by different T-cell subsets, with IgE inducing activities comparable with IL-4.[9,10]

The presence of IL-4 and IFN-γ has been demonstrated in cultured peripheral blood mononuclear cells (PBMC) from healthy individuals, atopic subjects and patients with the hyper-IgE syndrome.[11] Many studies have analysed cytokine production profiles of cultured and stimulated PBMC of allergic patients by ELISA,[12,13] or production of cytokines by cloned T cells.[14,15] However, only a few studies have described cytokine mRNA expression.[16,17] Ehlers *et al.* studied cytokine expression in neonatal T cells, compared with adult T cells, by PCR analysis. These authors showed that cord blood T cells are able, upon stimulation *in vitro*, to transcribe IL-2 mRNA. However, their capacity to transcribe mRNA for IL-3, IL-4, IL-5, IL-6, IFN-γ and GM–CSF was markedly reduced in comparison with that in adults.[16]

Our main interest is the role of cytokines in the development of allergy during infancy, since the immune system of children is generally considered to differ from the system in adults.[19] The number of T cells is relatively low and IFN-γ production by T-cells is decreased, in comparison with IFN-γ production in adults.[18,19]

We chose to analyse cytokine gene expression in young children (0–4 years), who are developing allergic asthma or atopic dermatitis, in order to study the pathogenesis of these diseases. We aim to correlate the mRNA expression levels in PBMC and T cells with the cytokine production profile and clinical manifestations (manuscript in preparation).

To make such studies feasible, T cells were purified from peripheral blood.[20] The amount of peripheral blood obtained from young children is usually small, and the number of T cells that can be purified is therefore limited. Moreover, cytokine gene expression is usually transient and cytokine mRNA levels generally occur at low abundance. Therefore, such analysis requires sensitive procedures.[21] The polymerase chain reaction (PCR)[22,23] is a sensitive method for the detection of gene expression. Here we describe optimizations of the PCR method to analyse cytokine gene expression occurring at low abundance and from a limited number of cells, in a semi-quantitative way.[24,25]

© 1995 Rapid Communications of Oxford Ltd

### Materials and Methods

*Purification and stimulation of T cells:* PBMC from a maximum of 4 ml heparinized blood of children, were purified by density centrifugation on Ficoll–Hypaque (Pharmacia, Uppsala, Sweden).[26] The children ranged from 0–4 years of age and were healthy or with allergic asthma. These PBMC were incubated (30 min, 4°C) with monoclonal antibodies specific for monocytes (My4, 100 μl (40 μg/ml) per 1 × $10^7$ pelleted cells, Coulter Cytometry, FL), B cells (CD19, 100 μl (100 μg/ml) per 1 × $10^7$ pelleted cells, Coulter) and natural killer cells (CD16 and CD56PE, both 50 μl (100 μg/ml) per 1 × $10^7$ pelleted cells, Becton Dickinson, Mountain View, CA). Subsequently, the samples were incubated (30 mins, 4°C on a roller bank) with sheep–anti-mouse Dynabeads (IgG M450, Dynal, Oslo, Norway). T cells were negatively selected by using a magnet (Dynal). The purity of the T-cell fraction was between 85% and 95%, as determined by FACS analysis, after staining with CD3-specific antibodies (Leu-4-FITC, 50 μl (2.5 μg/ml) per 5 × $10^5$ pelleted cells, Becton Dickinson). The contribution of natural killer cells was always less than 3.5%.

T cells (1 × $10^6$ cells/ml) were cultured for 16–18 h in Yssel's medium[27] containing 1% human serum with and without the addition of 4-bromo-calcium-ionophore (A23187, final concentration 500 ng/ml, Sigma, St. Louis, MO) and TPA (phorbol-12-myristate-13-acetate, final concentration 1 ng/ml, Sigma) at 37°C, 5% $CO_2$.

*RNA isolation and cDNA reaction:* After spinning down the T cells, RNA was isolated from the cells by the RNAzol B (Cinna-Biotecx Laboratories Inc., Houston, TX) method.[28] Briefly, per 1 × $10^6$ pelleted cells 200 μl RNAzol (minimum 300 μl) was added as well as chloroform (10 μl per 100 μl RNAzol B). After vigorous shaking and centrifugation, the aqueous phase was collected and an equal volume of phenol:chloroform (1:1) was added. After mixing and centrifugation, an equal volume of chloroform was added to the aqueous phase. Following centrifugation, an equal volume of isopropanol was added to the aqueous phase. After incubation at 4°C for at least 2 h, the RNA was pelleted by centrifugation and washed with 20 μl 70% ethanol per 1 × $10^6$ cells (minimum 50 μl). The RNA was resuspended in 10 μl water and the $OD_{260}/OD_{280}$ were determined by spectrophotometry (Ultrospec III, Pharmacia-LKB). Twenty μg glycogen (Boehringer Mannheim, Germany) was added during the phenol:chloroform extraction as a carrier.

cDNA synthesis[29] was performed starting with 1 μg RNA, after heating for 10 min at 65°C. Random hexamer primers were used to ensure all RNA was represented equally in the cDNA pool.[30] The reaction mixture contained 2 μl of 10× AMV–RT buffer (0.5 M Tris–HCl, 0.1 M $MgCl_2$, 0.5 M DTT, 10 mM EDTA and 100 μg/ml BSA; pH 8.3), 0.25 mM of each dNTP, 1 mM salmon spermine HCl (Sigma), 40 units of RNAsin (Promega, Madison, WI), 2.5 OD $(dN)_6$ (Pharmacia), 0.2 μg oligo$(dT)_{12-18}$ (Boehringer) and 5 units reverse transcriptase (from avian myeloblastosis virus; Boehringer). The total reaction volume was 20 μl. Incubation was performed for 1 h at 41°C. Afterwards the cDNA was diluted to 200 μl and stored at –70°C.

*Polymerase chain reaction:* For the polymerase chain reaction, a mixture was prepared containing 50 ng cDNA (in 10 μl) and 10 μl of 10× Taq polymerase buffer (100 mM Tris–HCl, 500 mM KCl, 15 mM $MgCl_2$ and 1% gelatin; pH 8.3), 0.03 mM of each dNTP and 1 unit of Taq polymerase (Ampli Taq, Perkin Elmer Cetus, Norwalk CT). One μl of the following sense- and anti-sense primers (10 OD/ml) were used:

| | | |
|---|---|---|
| *HPRT* | sense: | 5′-GTGATGATGAACCAGG-TTTATGACCTT-3′ (exon2), |
| | antisense: | 5′-CTTGCGACCTTGACCAT-CTTTGGA-3′ (exon 6), |
| | (product size 454 bp)[31] | |
| IL-4 | sense: | 5′-ACTCTGTGCACCGAGTT-GACCGTAA-3′ (exon 2), |
| | antisense: | 5′-TCTCATGATCTGCTTTAG-CCTTTCC-3′ (exon 4), |
| | (product size 300 bp)[32] | |
| IFN-γ | sense: | 5′-TTTAATGCAGGTCATTCA-GATG-3′ (exon 1–2) |
| | antisense: | 5′-CAGGGATGCTTCTTCGA-CCTCGAAAC-3′ (exon 4) |
| | (product size 388 bp)[32] | |

We developed the *HPRT* (human hypoxanthine phosphoribosyl transferase) primerset from the genomic structure, by selecting primers on exon 2 and 6. In this way, the product including the intron sequences will be too large (3.5 kB) to be amplified by PCR. The IL-4 and IFN-γ primersets were kindly donated by Dr R. de Waal Malefijt (DNAX Research Institute, Palo Alto, CA). It was verified by Southern blot analysis that both primersets did not amplify genomic DNA (data not shown).

The total reaction volume was 100 μl. As a negative control 50 ng RNA in 10 μl water, which had not undergone the cDNA reaction, was used from six patient samples per PCR. One sample of the cDNA mixture was also used as a negative control.

The PCR protocol started with an adaptation of the hot-start[33] to prevent aspecific primer annealing. After preparing the PCR mixture on ice, the reaction tubes were placed for 3 min in the PCR-block (DNA Thermal Cycler, Perkin–Elmer Cetus), which was preheated at 94°C. Subsequently, the first 25 PCR cycles (HPRT) were started: denaturation at 94°C for 0.5 min, annealing at 55°C for 0.5 min and extension at 72°C for 1 min. The linear phases of HPRT, IL-4 and IFN-γ were found during a different range of PCR cycles (data not shown). To cover these linear phases, the initial number of PCR cycles was 30 for IL-4 and 20 for IFN-γ. After 25 PCR cycles (HPRT) the block was cooled to 4°C, 10 μl of PCR product was removed from the reaction tubes and the tubes were taken out of the machine. Before re-introducing the reaction tubes, a hot-start was performed again, but now only for 1.5 min, to prevent too much loss of activity of the Taq enzyme. The half-life of Taq enzyme at 94°C is 35 min.[34] After five additional PCR cycles, 10 μl was collected again. This was repeated three times. The five time-point samples were analysed on agarose gel and subsequent Southern transfer.[35] The Southern transfer was performed to determine the specificity of the different primer sets and to ensure that no genomic DNA was amplified.

For analysis of patient samples, the procedure was ended by taking a photograph of the gel. B21 cDNA was used as a positive control in all PCR reactions, since the B21 Th0 clone produces among others IL-2, IL-4 and IFN-γ.[36] The B21 T-cell clone was kindly provided by Dr H. Yssel (DNAX Research Institute, Palo Alto, CA).

*Analysis of PCR products:* After the PCR amplification, the samples were loaded on a 1.2% agarose gel (Sea Kem LE agarose, FMC Bio Products, Rockland, ME), stained with 75 μg/ml ethidium bromide (Boehringer). One μl PhiX174 (HaeIII digest, 0.5 μg/μl, New England Biolabs, Beverly, MA) was used as a marker. The bands of the PCR products on the gel were visualised with UV light. A life-size photograph was taken and used for scanning. Each row (30 mm × 200 mm) of the photograph was scanned separately with a hand scanner (Colorscanner 2,[24] High-screen, Würselen, Germany) at a resolution of 100 dots per inch. Computer software was developed to analyse the intensity of the bands. A rectangle was drawn automatically around each band of a row. In each rectangle the intensity was measured on all 20 spots, from which the mean value was calculated by integrating the values. Two rectangles of the same size were drawn automatically directly next to the first row of rectangles. In this way the background was measured on two subsequent positions of the gel. After extrapolation, a correction could be made for a possible drift in the background staining of the gel. The mean background value was subtracted from the mean band value. The resulting value will be subsequently called the 'scan value'.

*Analysis of scan values:* The data collected with the hand scanner and the software were analysed in a spreadsheet (Quattro Pro for Windows, Borland International Inc., Scotts Valley, CA).

I. Calculation of the mean scan values of B21 cDNA for standardization of inter-experimental variation

HPRT, 35–40 cycles; IL-4, 40–45 cycles; IFN-γ, 30–35 cycles

II. On each gel with patient samples, one B21 sample run in the same PCR was applied. A correction factor could be calculated for B21, by dividing by the highest B21 scan value of the first three independent PCR runs, which were analysed on six different gels. Such B21 dependent correction was determined for every individual cytokine or HPRT

$$\frac{\text{B21 gel scan value HPRT/IL-4/IFN-}\gamma}{\text{highest B21 scan value HPRT/IL-4/IFN-}\gamma} = \text{B21 correction factor HPRT/IL-4/IFN-}\gamma$$

III. Correction of HPRT mRNA expression of a patient sample for B21 mRNA expression

$$\text{Scan value sample HPRT}^{*}\ \frac{1}{\text{B21 correction factor HPRT}}$$

IV. All patient scan value were corrected for HPRT by dividing by the highest HPRT scan value of five experiments, in order to correct for inter-patient variation

$$\frac{\text{HPRT scan value sample}}{\text{highest HPRT scan value}} = \text{HPRT correction factor}$$

V. Calculation of the mean values of HPRT of each patient sample after 35 and 40 PCR cycles

VI. Correction of IL-4 and IFN-γ mRNA expression for B21

$$\text{IL-4/IFN-}\gamma\text{ scan value sample*} \; \frac{1}{\text{B21 correction factor IL-4/IFN-}\gamma}$$

VII. Calculation of mean values of each sample

IL-4, 40–45 cycles and IFN-γ: 30–35 cycles

VIII. Calculation of the end values corrected for B21 and HPRT

$$\text{Mean sample value IL-4/IFN-}\gamma\text{*} \; \frac{1}{\text{correction factor HPRT}}$$

*Southern blotting:* Subsequently, the gel was put into an acid solution (0.25 M HCl) for 10 min, a denaturing solution (1 M NaCl, 0.5 M NaOH) twice for a period of 15 min, and a neutralizing solution (0.5 M Tris–HCl, 1.5 M NaCl, pH 7.0) twice for 20 min. After this a Southern transfer[35] was performed, by which the PCR products were transferred overnight to a Nytran filter (0.45 μm, Schleicher and Schuell, Dassel, Germany). DNA was crosslinked to the filter in a UV crosslinker (120 mJ) (UV Stratalinker 2400, Stratagene, La Jolla, CA).

Probes to the inner region of the amplification target were end-labelled with $^{32}$ATP (see Reference 37) and hybridization was carried out.[38] The following probes were used:

HPRT: 5′-GAAGAGCTATTGTAATGACCAGTCA-3′ (exon 3–4)[31]

IL-4: 5′-CAGTTCCACAGGCACAAGCAG-3′ (exon 3)[32]

IFN-γ: 5′-TGACTAATTATTCGGTAACTGACTTG-AATG-3′ (exon 3–4)[32]

### Results

*Semi-quantitative PCR analysis of cytokine gene expression:* Initially, three different housekeeping genes, HPRT[31], β-actin[39] and GAPDH (glyceraldehyde-3-phosphate dehydrogenase),[40] were tested on cDNA of different cell populations of healthy control children. The HPRT primer set was considered the most reliable, since it did not amplify genomic DNA, as tested with Southern blot analysis and hybridization. Furthermore, HPRT had a stable expression level in the same range as the cytokine levels studied (data not shown).

Next, T cells of a healthy control child were purified and cultured overnight in medium alone or stimulated polyclonally by the addition of Ca-ionophore and TPA. RT–PCR analysis was performed using primer sets specific for HPRT and IFN-γ. A representative result is shown in Fig. 1. After 25 cycles (HPRT) or 20 cycles (IFN-γ), 10 μl samples were taken from the PCR mixture and subsequently every five cycles up to 45 or 40 cycles, respectively. The PCR products were made visible by means of agarose gel electrophoresis.

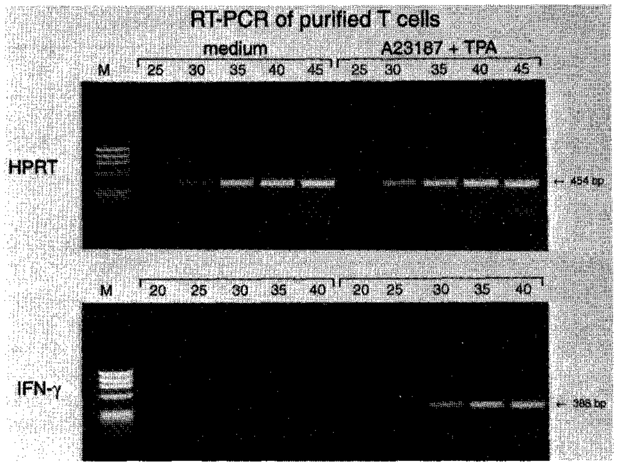

FIG. 1. RT–PCR of purified T cells. RNA was isolated from purified T cells of a healthy child. These cells had been cultured overnight in medium alone or stimulated with A23187 (Ca-ionophore) and TPA. After reverse transcription into cDNA, a PCR was performed for HPRT and IFN-γ. Samples were collected from the reaction tube after different numbers of cycles. For HPRT this was done at 25, 30, 35, 40 and 45 cycles and for IFN-γ at 20, 25, 30, 35, and 40 cycles. The HaeIII digest of PhiX174 was used as a molecular weight marker (M).

Visual analysis enabled qualitative observations like IFN-γ mRNA production by T cells after stimulation with Ca-ionophore and TPA. A differential mRNA expression was observed when comparing IFN-γ expression in T cells cultured in medium alone or stimulated with Ca-ionophore and TPA. The IFN-γ mRNA signal in the stimulated T-cell fraction was higher (already visible after 25 PCR cycles) than in the T-cell fraction cultured with medium alone (visible only after 35 cycles). In the same experiment, the HPRT mRNA appeared to be present in both samples in equal amounts.

It is possible to quantify these products by scanning the photographs and assigning values to the intensities of the different bands. To this end, the photograph of the agarose gel (Fig. 1) was scanned with a scanner and the image was analysed by the described software. The corresponding scan values are shown in Table 1. These results also reflect the differential expression shown in Fig. 1, but now in absolute numbers. For example, the scan values of the IFN-γ mRNA signal after 40 PCR cycles in stimulated T cells *vs*

**Table 1.** Scan values of HPRT and IFN-γ mRNA expression in purified T cells.

| | Number of cycles | | | | |
|---|---|---|---|---|---|
| | 25 | 30 | 35 | 40 | 45 |
| **HPRT** | | | | | |
| T cells medium | 0 | 349 | 1480 | 1753 | 1880 |
| T cells A23187 + TPA | 11 | 858 | 1643 | 1772 | 1763 |
| | Number of cycles | | | | |
| **IFN-γ** | 20 | 25 | 30 | 35 | 40 |
| T cells medium | 0 | 0 | 0 | 77 | 234 |
| T cells A23187 + TPA | 0 | 47 | 843 | 1048 | 1119 |

The photograph shown in Fig. 1 was scanned with the hand scanner and analysed by computer. Scan values are corrected for background.

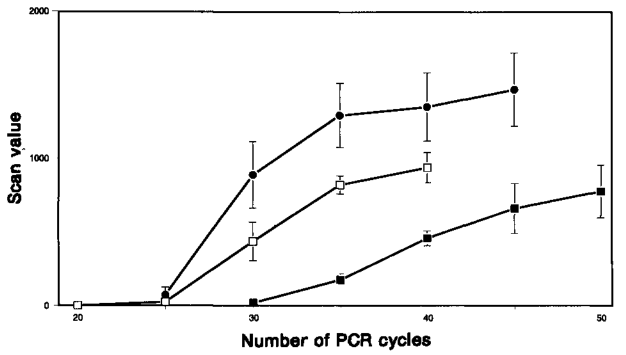

FIG. 2. Cytokine mRNA expression in stimulated B21 cells. cDNA of B21 was used for inter-experiment correction, using primers for HPRT (●), IL-4 (■) and IFN-γ (□). PCRs for HPRT were run from 25 to 45 cycles, for IL-4 from 30 to 50 cycles and for IFN-γ from 20 to 40 cycles, all with intervals of five cycles. The X-ordinate indicates the number of cycles. The Y-ordinate indicates the mean scan values. The data represent the mean of six different experiments ± S.D. of the obtained scan values.

T cells cultured in medium alone, were 1048 compared with 77 (a 13.6-fold increase). Both types of analyses show that the PCR reaction for HPRT reached a plateau phase after 40 PCR cycles. For IFN-γ a plateau level was reached after 35 PCR cycles, but only in the stimulated T-cell fraction.

*Linearity of the PCR:* It is crucial to compare the results in the linear face of the PCR,[24] in order to permit comparison in cytokine mRNA expression between different patients and patient groups. Therefore, we tested which part of the PCR for HPRT and the relevant cytokine genes was linear. To this end, one large batch of cDNA of the B21 T-cell clone was used in the PCR for HPRT, IL-4 and IFN-γ. In Fig. 2 the mean scan values are shown of B21 cDNA run in 3 independent PCR reactions, while the PCR products were analysed on six different agarose gels. The linear phase of the HPRT PCR was consistently found between 25 and 35 cycles, after which a plateau level was reached. Similar findings were made for IFN-γ. The linear phase of the IL-4 PCR was found between 35 and 45 cycles. Thus, different samples can only be compared by analysing data obtained at these predetermined numbers of PCR cycles.

*Standardization of differences in cytokine mRNA production between different patients:* HPRT was used as a housekeeping gene to standardize the cytokine mRNA expression of each patient, enabling analysis of inter-patient variation within the PCR reaction. However, for obvious reasons it was not possible to perform all the PCR reactions at the same time. Therefore, an inter-experiment standard was considered necessary. To this end the B21 T-cell clone was used. This clone produced, among others, the cytokines IL-4 and IFN-γ.[36] From a large number of B21 cells, RNA was isolated and transcribed into cDNA. Aliquots of this batch were used for all PCR experiments. Fig. 2 shows the kinetics of the PCR reaction of B21 cDNA. HPRT and IFN-γ reached a plateau relatively early (HPRT after 35 PCR cycles and IFN-γ after 40), while the linear phase of IL-4 was longer (plateau only after 45–50 PCR cycles). The variation in B21 scan values, obtained from independent PCR runs and analysed on different gels, was relatively small (less than 10%), resulting in a generally small inter-experiment variation.

*Analysis of the scan values:* To optimize the reproducibility of data, photographs were processed in a similar way. Special attention was paid to obtaining gel pictures of the same intensity, contrast and paper quality. The reproducibility of the data generated with the scanner and the computer software were also examined. On a photograph displaying amplified PCR products one lane was scanned ten times. The results are depicted in Table 2. The variation in the data collected was always less than 10%.

As described in 'linearity of the PCR', comparisons have to be made in the linear phase of the reaction. To correct for possible fluctuations, mean scan values were calculated from the two PCR cycle times in the linear phase of the PCR. This phase was determined for each gene studied.

Correction factors were calculated for B21 mRNA. The mean correction factor for B21 HPRT was 0.78 (± 0.12), for B21 IL-4 0.75 (± 0.14) and for B21 IFN-γ 0.74 (± 0.14). The relatively small standard deviations show that the repro-

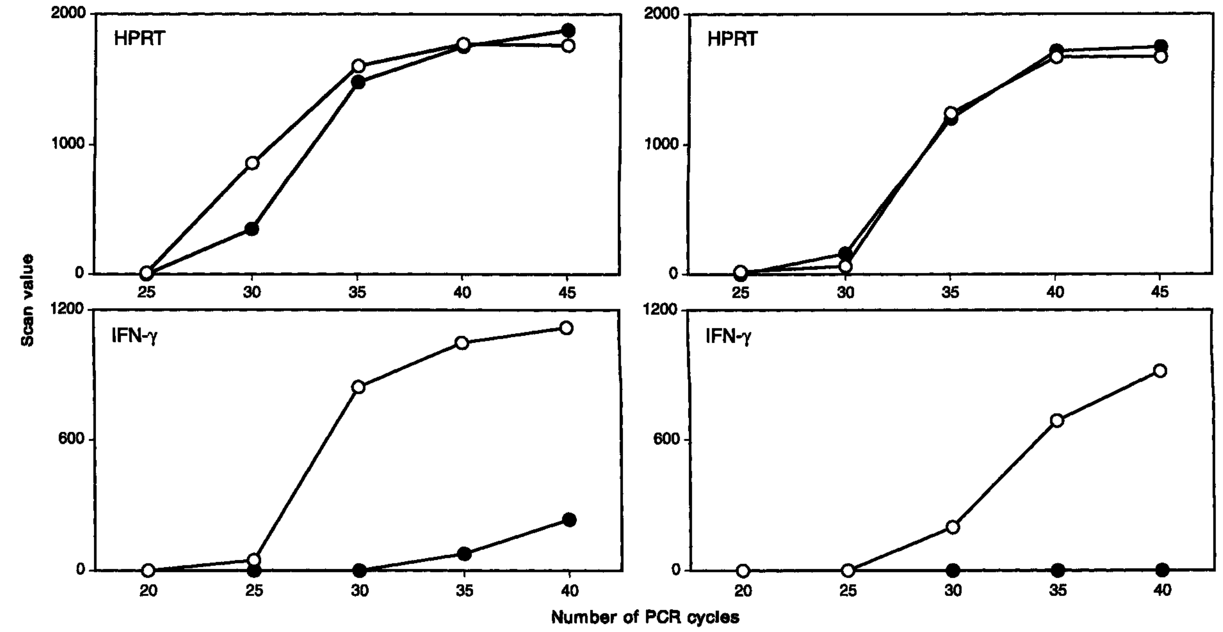

FIG. 3. Analysis of HPRT and IFN-γ mRNA expression in a healthy and an asthmatic child. T cells were purified from peripheral blood of a healthy and an asthmatic child. T cells were cultured overnight in medium alone (●) or with the addition of A23187 (Ca-ionophore) and TPA (○). RT-PCR was performed for: a healthy child for HPRT (upper left), an asthmatic child for HPRT (upper right), a healthy child for IFN-γ (lower left) and an asthmatic child for IFN-γ (lower right).

**Table 2.** Reproducibility of the hand scanner

| Band | Mean | S.D. | *n* |
|---|---|---|---|
| 1 | 665.2 | 43.57 | 10 |
| 2 | 1380.8 | 98.43 | 10 |
| 3 | 1514.2 | 169.35 | 10 |

One lane of an 1.2% agarose gel with 75 μg/500 ml ethidium bromide was scanned ten times. Arithmetic mean and S.D. were calculated for three bands with different levels of intensity.

ducibility of the PCR of B21 cDNA was high. For this reason, only small inter-experiment corrections (mean 0.77 ± 0.14) were generally necessary. The mean correction factor for HPRT mRNA expression of 20 samples was 0.81 ± 0.21. In case the correction factors for B21 and HPRT were smaller than 0.5, or greater than 1.5, we considered the data not to be reliable because of possible overcorrection. The results of such experiments (in the case of B21) or samples (in the case of HPRT) were excluded from further analysis.

*Application of the technique to patient samples:* T cells were purified from peripheral blood of healthy and asthmatic children. The T cells were cultured overnight in medium alone or stimulated with a Ca-ionophore and TPA. After RNA isolation and cDNA synthesis, PCR was performed for HPRT, IL-4 and IFN-γ during the described number of PCR cycle times. In Fig. 3 a representative example is shown of one healthy child and one asthmatic child. The shapes of the curves for HPRT were comparable, both for T cells cultured in medium and for stimulated T cells. All curves reached a similar plateau level (1766 ± 74). In both the healthy control (lower left) and the asthmatic child (lower right) there was a marked difference in IFN-γ mRNA expression between stimulated and unstimulated T cells. In stimulated T cells from the healthy control, the plateau phase of IFN-γ PCR was reached earlier (35 PCR cycles) than in the stimulated T cells of the asthmatic child (40 PCR cycles), indicating a higher expression of IFN-γ mRNA. In the unstimulated condition, no IFN-γ expression was found in the T cells from the asthmatic child, whereas after 35 and 40 PCR cycles IFN-γ expression could be detected in T cells from the healthy control. The linear range of the PCR as assessed in B21 cells, was also applicable to HPRT and cytokine gene expression in patient samples of stimulated and unstimulated T cells.

In Table 3 the correction method is applied on the scan values of the healthy and asthmatic children presented in Fig. 3. First, the inter-experiment variation was determined by calculating the B21 correction factors for HPRT and IFN-γ. As shown in Table 3, the inter-experiment correction factor for HPRT was similar for the healthy and the asthmatic child samples (0.76 *vs*

**Table 3.** Correction factors for HPRT and IFN-γ for B21 clone

| Subjects | B21 correction factor | |
|---|---|---|
| | HPRT | IFN-γ |
| Healthy | 0.76 | 0.53 |
| Asthma | 0.70 | 0.99 |

Correction factors were calculated for B21 mRNA expression of HPRT and IFN-γ, as described in 'Analysis of scan values'.

**Table 4.** HPRT correction factors and IFN-γ end values for purified T cells

| Subjects | Correction factor HPRT | | End values IFN-γ | |
|---|---|---|---|---|
| | Control | Stimulation | Control | Stimulation |
| Healthy | 1.01 | 1.06 | 71.31 | 1673.69 |
| Asthma | 0.99 | 0.99 | 0.00 | 444.68 |

Correction factors were calculated for purified T cells of a healthy and an asthmatic child for HPRT mRNA expression and end values were calculated for IFN-γ, as described in 'Analysis of scan values'. T cells were cultured overnight in medium (control) or stimulated with TPA and Ca-ionophore (stimulation).

0.70). For IFN-γ this correction factor differed more (0.53 *vs* 0.99). For the subsequent inter-patient correction, the HPRT correction factors were minimal, as shown in Table 4. The end values for IFN-γ could be calculated with these correction factors (Table 4). The qualitative differences as observed in Fig. 3 were also reflected in these end values, but now in a semi-quantitative way (1673.69 *vs* 444.68). This analysis allows comparison of cytokine expression between different cell populations of larger patient groups.

### Discussion

Our studies have shown that semi-quantitative analysis of cytokine gene expression is possible in small peripheral blood samples. This was achieved by adapting the RT–PCR method and combining it with an easy and reliable way of analysing the amplified product. For small numbers of cells, other methods of studying cytokine gene expression, such as Northern blot analysis, cannot be employed because of the low amount of isolated RNA. PCR enables analysis of gene expression in a highly sensitive and specific way.[41]

Several adaptations of the PCR procedure were necessary to permit the required analysis. The analysis of the expression of a housekeeping gene is generally considered essential to correct for inter-patient variation.[42] The requirements for a suitable housekeeping gene are a stable expression, a similar detection range as the expression of the genes to be studied and no amplification of genomic DNA.[43] A HPRT primer set was developed, which meets the above criteria as verified by Southern blot analysis and hybridization. DNAse treatment,[44] often used when RNA is contaminated with genomic DNA, resulted in a significant loss of RNA and could therefore not be used in our studies.

Determining the linear phase of the PCR was important because at the plateau phase the quantified amount of amplified product is no longer proportional to the starting amount of target molecules.[24,25] The PCR is linear only during a limited number of cycles, after which the amount of product reaches a plateau.[24] There are a number of factors that contribute to this plateau phenomenon, including substrate saturation of enzyme, product strand reannealing and incomplete product strand separation.[25] The linear phase of the PCR has to be determined for each gene to be analysed, because the primers used to amplify their products differ in G/C versus A/T content and thus in annealing temperatures.

Preliminary experiments indicated that the described approach could also be applied to the analysis of PCR products, that are detected by autoradiography. This way the sensitivity of the analysis was further increased. Therefore, the algorithm developed here can analyse films of Southern blots, as was found necessary for IL-5 and IL-10.

Visual analysis of the photographed gels enabled us to make qualitative comparisons between different patient samples. However, the appearance of a positive signal after a certain number of cycles did not necessarily reflect the original mRNA concentration of the relevant gene. Two different samples could reach a detectable PCR signal after the same number of PCR cycles, while the intensity of the bands was different and another plateau level was reached. Therefore, quantitative analysis of the bands was considered necessary. This was achieved by using scanning in combination with newly developed software for data analysis.

Another necessary step in the standardization of the procedure was the use of B21, a Th0 clone, which produces upon activation all cytokines relevant for this study.[36] B21 cDNA gave reproducible data for HPRT, IL-4 and IFN-γ expression in different experiments. The inter-experiment correction was thus generally small (0.77 ± 0.14). This correction had to be determined for every individual cytokine. The variation of HPRT from different patient samples was in the same range (0.81 ± 0.21) as for B21, therefore a similar inter-patient correction was necessary. When the variation is too large, over-correction can occur. We consider a correction factor smaller than 0.5 or larger than 1.5 too extreme to detect the cytokine response in the sample reliably.

The analysis developed here is a reliable and simple alternative to other methods based on the use of radioactivity, like phosphor imaging.[45] The variation found in cytokine expression within a patient group of, for example, healthy children of the same age is relatively high (Koning *et al.*, manuscript in preparation). The accuracy of the semi-quantitative method described here has sufficient discernment in relation to this extent of variation.

Recently, true quantitative methods have been developed. The method developed by Gilliland *et al.*[46] uses an internal standard with the same primer requirement, but differing in the size of PCR product. However, the accuracy of quantification may be affected by sequence differences between the DNAs used for the standard and the sample.[47]

When studying the intrinsic capacity of cells to express a particular cytokine gene, it is necessary to analyse highly purified cell populations. However, many studies examine the significance of cytokines in patients by analysing the total PBMC fraction only.[11,13] When endogenous cytokine gene expression is to be studied in purified T cells, it is important to note that the T cells should not be stimulated during purification. Indeed, negative selection during purification of T cells was found to be adequate to avoid stimulation. It is also important that for some cytokines, e.g., IFN-γ, cells need to be stimulated to obtain detectable expression levels. In our study, we used a polyclonal stimulus, Ca-ionophore and TPA, permitting the simultaneous detection of a broad range of cytokines. For such studies allergen specific stimulation can also be applied (manuscript in preparation).

We conclude that it is possible to accurately detect differential cytokine gene expression in T cells isolated from blood of healthy controls and asthmatic children. This can be achieved by using a RT–PCR with differential cycle times for different cytokines and analysing the results with a scanner and specially designed computer software. Cytokine gene expression can thus be studied in a very limited amount of material.

### References

1. Leung DYM. Role of IgE in atopic dermatitis. *Curr Opin Immunol* 1993; **5**: 956–962.
2. Corrigan CJ. Allergy of the respiratory tract. *Curr Opin Immunol* 1992; **4**: 798–804.
3. Pène J. Heterogeneity of atopy demonstrated by differences in cytokine release by peripheral blood T cells. *Ann Allergy* 1993; **71**: 322–326.
4. de Vries JE, Gauchat J-F, Aversa G, Punnonen J, Gascan H, Yssel H. Regulation of IgE synthesis by cytokines. *Curr Opin Immunol* 1991; **3**: 851–858.
5. Gauchat J-F, Lebman DA, Coffman RL, Gascan H, de Vries JE. Structure and expression of germline-ε transcripts in human B cells induced by interleukin-4 to switch to IgE production. *J Exp Med* 1990; **172**: 463–473.
6. Pène J, Rousset F, Brière F, *et al.* IgE production by human B cells is induced by interleukin-4 and suppressed by interferons gamma, alpha and prostaglandin $E_2$. *Proc Natl Acad Sci* USA. 1988; **85**: 6880–6884.
7. Corrigan CJ, Haczku A, Gemou-Engesaeth V, *et al.* CD4 T-lymphocyte activation in asthma is accompanied by increased serum concentrations of Interleukin-5. *Am Rev Resp Dis* 1993; **147**: 540–547.
8. Moore KW, O'Garra A, de Waal Malefyt R, Vieira P, Mossmann R. Interleukin-10. *Ann Rev Immunol* 1993; **11**: 165–190.
9. Minty A, Chalon P, Derocq J, *et al.* Interleukin-13 is a new lymphokine regulating inflammatory and immune responses. *Nature* 1993; **362**: 248–250.
10. Punnonen J, Aversa G, Cocks BG, *et al.* Interleukin 13 induces interleukin 4-independent IgG4 and IgE synthesis and CD23 expression by human B cells. *Proc Natl Acad Sci* 1993; **90**: 3730–3734.
11. Rousset F, Robert JR, Andary M, *et al.* Shifts in interleukin-4 and interferon-γ production by T cells of patients with elevated serum IgE levels and the modulatory effects of these lymphokines on spontaneous IgE synthesis. *J Allergy Clin Immunol* 1991; **87**: 58–69.
12. Jujo K, Renz H, Abe J, Gelfland E, Leung M. Decreased interferon gamma and increased interleukin-4 production in atopic dermatitis promotes IgE synthesis. *J Allergy Clin Immunol* 1992; **90**: 323–331.
13. Tang M, Kemp A, Vangos G. IL-4 and interferon-gamma production in children with atopic disease. *Clin Exp Immunol* 1993; **92**: 120–124
14. Holt PG, Clough JB, Holt BJ, *et al.* Genetic 'risk' for atopy is associated with delayed postnatal maturation of T-cell competence. *Clin Exp Allergy* 1992; **22**: 1093–1099.
15. Wierenga EA, Snoek MA, De Groot C, *et al.* Evidence for compartimentalization of functional subsets of $CD4^+$ T-lymphocyte in atopic patients. *J Immunol* 1990; **144**: 4651–4656.
16. Ehlers S, Smith KA. Differentiation of T cell lymphokine gene expression: the *in vitro* acquisition of T cell memory. *J Exp Med* 1991; **173**: 25–36.
17. Huang S-K, Essayan DM, Krishnaswamy G, *et al.* Detection of allergen- and mitogen-induced human cytokine transcripts using a competitive polymerase chain reaction. *J Immunol Methods* 1994; **168**: 167–181.
18. Holt PG, McMenamin C, Nelson D. Primary sensitisation to inhalant allergens during infancy. *Ped Allergy Immunol* 1990; **1**: 3–13.
19. Wilson CB, Westall J, Johnston L, Lewis DB, Dower SK, Alpert AR. Decreased production of interferon-gamma by human neonatal cells. *J Clin Invest* 1986; **77**: 860–867.
20. Baert MRM, Koning H, Neijens HJ, Oranje AP, de Groot R, Savelkoul HFJ. The role of the immune system in allergic children. *Ped Allergy Immunol* 1995; **6** suppl.
21. Dallman MJ, Montgomery RA, Larsen CP, Wanders A, Wells AF. Cytokine gene expression: analysis using Northern blotting, polymerase chain reaction and *in situ* hybridization. *Immunol Reviews* 1991; **119**: 163–179.
22. Mullis KB, Faloona F. Specific synthesis of DNA *in vitro* via a polymerase catalyzed chain reaction. *Methods Enzymol* 1987; **155**: 335–350.
23. Erlich HA, Gelflad D, Sninsky JJ. Recent advances in the polymerase chain reaction. *Science* 1991; **252**: 1643–1651.
24. Ferre F. Quantitative or semi-quantitative PCR: reality versus myth. *PCR Meth and Appl* 1992; **2**: 1–9.
25. Bell J. The polymerase chain reaction. *Immunol Today* 1989; **10**: 351–355.
26. Böyum A. Separation of leukocytes from blood and bone marrow. *Scan J Clin Lab Invest* 1968; **97**(suppl 21): 7.
27. Yssel H, de Vries JE, Koken M, van Blitterswijk W and Spits H. Serum-free medium for generation and propagation of functional human cytotoxic and helper T cell clones. *J Immunol Methods* 1984; **72**: 219–227.
28. Chromczynski P, Sacchi N. Single-step method of RNA isolation by acid guanidinium thiocyanate–phenol–chloroform extraction. *Anal Biochem* 1986; **162**: 156–159.
29. Krug M, Berg S. First strand cDNA synthesis primed with oligo(dT). *Methods Enzymol* 1987; **152**: 316–325.
30. Tan S, Weis J. Development of a sensitive reverse transcriptase PCR assay, RT–PCR, utilizing rapid cycle times. *PCR Meth and Appl* 1992; **2**: 137–143.
31. Jolly DJ, Okayama H, Berg P, *et al.* Isolation and characterization of a full-length expressible cDNA for human hypoxanthine phosphoribosyltransferase. *Proc Natl Acad Sci* 1983; **80**: 477–481.
32. de Waal Malefijt, R. Molecular aspects of human T cell activation and lymphokine production. Thesis, Amsterdam, 1991, p. 201.
33. Mullis, KB. The polymerase chain reaction in an anemic mode: how to avoid cold oligodeoxyribonuclear fusion. *PCR Meth and Appl* 1991; **1**: 1–4.
34. Sardelli A. Plateau effect — understanding PCR limitations. *Amplifications* 1993; **9**: 1–5.
35. Maniatis T, Fritsch EF, Sambrook J. Southern transfer. In: Ford N, Nolan

C, Ferguson M, eds. *Molecular Cloning, a Laboratory Manual*, New York, Cold Spring Harbor Laboratory, 1989; 9.31–9.37.

36. Paliard X, de Waal Malefijt R, Yssel H, *et al.* Simultaneous production of IL-2, IL-4 and IFN-γ by activated human CD4$^+$ and CD8$^+$ T cell clones. *J Immunol* 1988; **141**: 849–855.
37. Tabor S. 5'End labeling with T4 polynucleotide kinase. In: Ford N, Nolan C, Ferguson M, eds. *Current Protocols in Molecular Biology.* Wiley, New York; 1990; **1**: 3.10.2.–3.10.5.
38. Strauss WN. Hybridization with radioactive probes. In: Ford N, Nolan C, Ferguson M, eds. *Current Protocols in Molecular Biology* Wiley, New York; 1990; **1**: 6.4.1.–6.4.10.
39. Nakajima-Iijima S, Hamada H, Reddy P, Kakunaga T. Molecular structure of the human cytoplasmatic β-actin gene: interspecies homology of sequences in the introns. *Proc Natl Acad Sci* 1985; **82**: 6133–6137.
40. Ercolani L, Florence B, Denaro M, Alexander M. Isolation and complete sequence of a functional human glyceraldehyde-3-phosphate dehydrogenase gene. *J. Biol Chem* 1988; **263**: 15335–15341.
41. Wang AM, Doyle MV, Mark DF. Quantitation of mRNA by the polymerase chain reaction. *Proc Natl Acad Sci* 1989; **86**: 9717–9721.
42. Chelly J, Kaplan J-C, Marie P, Gaustron S, Kahan A. Transcription of the distrophin gene in human muscle and non muscle tissues. *Nature* 1988; **333**: 858–860.
43. O'Garra A, Vieira P. Polymerase chain reaction for detection of cytokine gene expression. *Curr Opin Immunol* 1992; **4**: 211–215.
44. Sun L, Pettinger WA. Removal of genomic DNA for RNA PCR. *Focus.* 1993; **15**: 70–71.
45. Patterson SD, Latter GI. Evaluation of storage phosphor imaging for quantitative analysis of 2-D gels using the Quest II system. *Biotechniques* 1993; **15**: 1076–1083.
46. Gilliland G, Perrin S, Blanchard K, Bunn FH. Analysis of cytokine mRNA and DNA: detection and quantitation by competitive polymerase chain reaction. *Proc Natl Acad Sci* 1990; **87**: 2725–2729.
47. Dallman M, Porter A. Semi-quantitative PCR for the analysis of gene expression. In: McPherson MJ, Quirke P, Taylor GR, eds. *PCR a practical approach* Oxford: IRL Press, 1991; 215–224.

ACKNOWLEDGEMENTS. We thank Drs H. J. Imbens for developing the computer software for analysing the gels; J. Landegent for initial discussions; and T. R. Rinke de Wit and R. Benner for critically reviewing this manuscript and making helpful suggestions. This work was partially supported by a grant from the Glaxo Research Fund, Netherlands.

**Received 14 February 1995; accepted 8 March 1995**

## References

[PDF_00824] Chelly J., Kaplan J. C., Maire P., Gautron S., Kahn A. (1988). Transcription of the dystrophin gene in human muscle and non-muscle tissue.. Nature.

[PDF_00729] Corrigan C. J., Haczku A., Gemou-Engesaeth V., Doi S., Kikuchi Y., Takatsu K., Durham S. R., Kay A. B. (1993). CD4 T-lymphocyte activation in asthma is accompanied by increased serum concentrations of interleukin-5. Effect of glucocorticoid therapy.. Am Rev Respir Dis.

[PDF_00755] Ehlers S., Smith K. A. (1991). Differentiation of T cell lymphokine gene expression: the in vitro acquisition of T cell memory.. J Exp Med.

[PDF_00819] Ercolani L., Florence B., Denaro M., Alexander M. (1988). Isolation and complete sequence of a functional human glyceraldehyde-3-phosphate dehydrogenase gene.. J Biol Chem.

[PDF_00722] Gauchat J. F., Lebman D. A., Coffman R. L., Gascan H., de Vries J. E. (1990). Structure and expression of germline epsilon transcripts in human B cells induced by interleukin 4 to switch to IgE production.. J Exp Med.

[PDF_00834] Gilliland G., Perrin S., Blanchard K., Bunn H. F. (1990). Analysis of cytokine mRNA and DNA: detection and quantitation by competitive polymerase chain reaction.. Proc Natl Acad Sci U S A.

[PDF_00749] Holt P. G., Clough J. B., Holt B. J., Baron-Hay M. J., Rose A. H., Robinson B. W., Thomas W. R. (1992). Genetic 'risk' for atopy is associated with delayed postnatal maturation of T-cell competence.. Clin Exp Allergy.

[PDF_00792] Jolly D. J., Okayama H., Berg P., Esty A. C., Filpula D., Bohlen P., Johnson G. G., Shively J. E., Hunkapillar T., Friedmann T. (1983). Isolation and characterization of a full-length expressible cDNA for human hypoxanthine phosphoribosyl transferase.. Proc Natl Acad Sci U S A.

[PDF_00788] Krug M. S., Berger S. L. (1987). First-strand cDNA synthesis primed with oligo(dT).. Methods Enzymol.

[PDF_00713] Leung D. Y. (1993). Role of IgE in atopic dermatitis.. Curr Opin Immunol.

[PDF_00797] Mullis K. B. (1991). The polymerase chain reaction in an anemic mode: how to avoid cold oligodeoxyribonuclear fusion.. PCR Methods Appl.

[PDF_00816] Nakajima-Iijima S., Hamada H., Reddy P., Kakunaga T. (1985). Molecular structure of the human cytoplasmic beta-actin gene: interspecies homology of sequences in the introns.. Proc Natl Acad Sci U S A.

[PDF_00807] Paliard X., de Waal Malefijt R., Yssel H., Blanchard D., Chrétien I., Abrams J., de Vries J., Spits H. (1988). Simultaneous production of IL-2, IL-4, and IFN-gamma by activated human CD4+ and CD8+ T cell clones.. J Immunol.

[PDF_00831] Patterson S. D., Latter G. I. (1993). Evaluation of storage phosphor imaging for quantitative analysis of 2-D gels using the Quest II system.. Biotechniques.

[PDF_00737] Punnonen J., Aversa G., Cocks B. G., McKenzie A. N., Menon S., Zurawski G., de Waal Malefyt R., de Vries J. E. (1993). Interleukin 13 induces interleukin 4-independent IgG4 and IgE synthesis and CD23 expression by human B cells.. Proc Natl Acad Sci U S A.

[PDF_00715] Pène J. (1993). Heterogeneity of atopy demonstrated by differences in cytokine release by peripheral blood T cells.. Ann Allergy.

[PDF_00726] Pène J., Rousset F., Brière F., Chrétien I., Bonnefoy J. Y., Spits H., Yokota T., Arai N., Arai K., Banchereau J. (1988). IgE production by normal human lymphocytes is induced by interleukin 4 and suppressed by interferons gamma and alpha and prostaglandin E2.. Proc Natl Acad Sci U S A.

[PDF_00790] Tan S. S., Weis J. H. (1992). Development of a sensitive reverse transcriptase PCR assay, RT-RPCR, utilizing rapid cycle times.. PCR Methods Appl.

[PDF_00822] Wang A. M., Doyle M. V., Mark D. F. (1989). Quantitation of mRNA by the polymerase chain reaction.. Proc Natl Acad Sci U S A.

[PDF_00752] Wierenga E. A., Snoek M., de Groot C., Chrétien I., Bos J. D., Jansen H. M., Kapsenberg M. L. (1990). Evidence for compartmentalization of functional subsets of CD2+ T lymphocytes in atopic patients.. J Immunol.

[PDF_00782] Yssel H., De Vries J. E., Koken M., Van Blitterswijk W., Spits H. (1984). Serum-free medium for generation and propagation of functional human cytotoxic and helper T cell clones.. J Immunol Methods.

[PDF_00719] de Vries J. E., Gauchat J. F., Aversa G. G., Punnonen J., Gascan H., Yssel H. (1991). Regulation of IgE synthesis by cytokines.. Curr Opin Immunol.

